# Ethanolamine and Vinyl–Ether Moieties in Brain Phospholipids Modulate Behavior in Rats

**DOI:** 10.3390/neurosci5040037

**Published:** 2024-11-04

**Authors:** MST Zenika Nasrin, Shuhei Kikuchi, Yasuhiro Uchimura, Mina Yoshioka, Shin-ya Morita, Tomoya Kobayashi, Yusuke Kinoshita, Yoshio Furusho, Hitoshi Tamiaki, Daijiro Yanagisawa, Jun Udagawa

**Affiliations:** 1 Division of Anatomy and Cell Biology, Department of Anatomy, Shiga University of Medical Science, Otsu 520-2192, Shiga, Japan; zenikabmb12@gmail.com (M.Z.N.); kikuchii@belle.shiga-med.ac.jp (S.K.); uchimura@belle.shiga-med.ac.jp (Y.U.);; 2 Department of Pharmacotherapeutics, Shiga University of Medical Science, Otsu 520-2192, Shiga, Japan; 3 Graduate School of Life Sciences, Ritsumeikan University, Kusatsu 525-8577, Shiga, Japan; 4 Department of Chemistry, Shiga University of Medical Science, Otsu 520-2192, Shiga, Japan; 5 Molecular Neuroscience Research Center, Shiga University of Medical Science, Otsu 520-2192, Shiga, Japan

**Keywords:** phospholipids, plasmalogen, behavior, locomotor activity, memory

## Abstract

Plasmalogens are brain-enriched phospholipids with a vinyl–ether bond at the *sn*-1 position between the glycerol backbone and the alkyl chain. Previous studies have suggested that plasmalogens modulate locomotor activity, anxiety-like behavior, and cognitive functions in rodents; however, the specific moieties contributing to behavioral regulation are unknown. In this study, we examined the behavioral modulation induced by specific phospholipid moieties. To confirm the permeability of phospholipids in injected liposomes, we measured the fluorescence intensity following intravenous injection of liposomes containing ATTO 740-labeled dioleoylphosphatidylethanolamine. Then, we compared the behavioral effects following injection of liposomes composed of egg phosphatidylcholine (PC) and 1-stearoyl-2-docosahexaenoyl-*sn*-glycero-3-phosphoethanolamine (PE 18:0/22:6), PC 18:0/22:6, 1-(1Z-octadecenyl)-2-docosahexaenoyl-*sn*-glycero-3-phosphoethanolamine (PE P-18:0/22:6), or PC P-18:0/22:6, into the tail vein of male rats. The time spent in the central region of the open field was significantly reduced after injection of PE 18:0/22:6, harboring an ester bond at *sn*-1 compared to controls. Furthermore, the discrimination ratio in the novel object recognition test was significantly higher in PC 18:0/22:6 compared to PE 18:0/22:6, suggesting that the substitution of ethanolamine with choline can enhance recognition memory. We demonstrate that the structures of the *sn*-1 bond and the hydrophilic moiety in the phospholipids can modulate exploratory behaviors and recognition memory in rodents.

## 1. Introduction

Glycerophospholipids (GPs) are amphipathic molecules composed of a hydrophilic moiety in the head group linked to hydrophobic moieties, such as an acyl, alkyl ether, or alkenyl ether chain, through a glycerol backbone. Among the GPs, phosphatidylcholine (PC) and phosphatidylethanolamine (PE) are common constituents of biological membranes [1], and plasmalogens, which have a vinyl–ether bond at the *sn*-1 position, constitute approximately 15–20% of the total phospholipids in the cell membrane [2]. Plasmalogens are enriched in the brain, with ethanolamine plasmalogen (PlsEtn) accounting for over 50% of the total PE in gray matter and over 80% in myelin [2,3]; hence, plasmalogens are critical structural components of neural cells. Membrane lipids are also important substrates for intracellular transduction pathways that modulate core brain functions such as development, plasticity, and cognition, as well as pathological processes such as neuroinflammation and neurodegeneration. Plasmalogen deficiency has been implicated in the pathology of Alzheimer’s disease (AD) and the development of rhizomelic chondrodysplasia punctata, which are associated with disturbances in brain structure and function [4,5,6,7]. Plasmalogens also modulate behavior, as plasmalogen-deficient mice have been shown to exhibit memory impairments and hyperactivity, while oral administration of PlsEtn rescued memory impairments and restored normal behavior in rodent models of neurological disease [8,9,10].

The neuroregulatory functions of plasmalogens are dependent on specific bio-physical and chemical properties conferred by key moieties [11]. For instance, phosphoethanolamine in the hydrophilic moiety and the vinyl–ether bond at the *sn*-1 position contribute to the formation of a hexagonal II phase that increases lipid bilayer rigidity by inducing more ordered acyl chains in the hydrophobic portions [12]. These properties can, in turn, alter membrane trafficking and impair synaptic transmission. Altered plasmalogen composition in membrane lipid rafts also modulates proinflammatory signaling in the hippocampus [8]. In addition to modulating the membrane phase, fluidity, and rigidity, the vinyl–ether bond in plasmalogens serves as a free radical scavenger; in fact, PlsEtn has been reported to alleviate amyloid β-induced neuro-toxicity through inhibiting oxidative stress [13]. Further, plasmalogens serve as reservoirs for polyunsaturated fatty acids (PUFAs) such as docosahexaenoic acid (DHA), which has anti-inflammatory and anti-apoptotic effects, and arachidonic acid (AA), which is pro-inflammatory [11]. The hydrophilic moieties in phospholipids may also possess unique functional properties which are important for neural signaling. Indeed, a recent in vitro study has demonstrated a protective effect of choline plasmalogen-oleic acid (PlsCho-OA)—but not PlsEtn-OA, AA, DHA, or PlsCho-AA—on AA-induced cytotoxicity of SY5Y human neuroblastoma cells [14]. PC has also been reported to rescue anxiety-like behavior and reduced social preference in Mecp2-conditional knockout mice, a model of Rett syndrome [15], while plasma ether-linked PC levels were inversely correlated with the severity of melancholia in major depressive disorder patients [16]. Accordingly, it is conceivable that the ether bond at the *sn*-1 position and PUFAs at the *sn*-2 position (as well as plasmalogen hydrophilic moieties) have distinct functions related to neuroprotection and behavioral regulation; however, the specific functions of individual moieties or combinations of moieties have not yet been demonstrated.

To examine the characteristic effects of specific hydrophilic and hydrophobic phospholipid moieties and *sn*-1 position bonds in plasmalogens on rodent behavior, we injected rats with the liposomes designed to deliver the phospholipids through the blood-brain barrier (BBB) and altered brain lipid profiles. The augmentation of specific phospholipids through the administration of liposomes may be a promising strategy for the treatment of neurodevelopmental, psychiatric, and neurodegenerative disorders.

## 2. Materials and Methods

### 2.1. Animals

All the animal procedures were approved by the Shiga University of Medical Science Animal Care and Use Committee (2022-8-5, 2022-9-5). For experiments investigating the incorporation of PE into the brain, we used 7-week-old male ICR mice obtained from CLEA Japan, Inc. (Tokyo, Japan). For experiments examining the effects of phospholipid-containing liposomes on behavior, we used 9-week-old male Wistar rats obtained from CLEA Japan. All the mice and rats were housed at room temperature (2–25 °C) under 40–60% relative humidity and a 12 h:12 h light: dark cycle (lights on at 08:00). All the animals were fed CE-2 ad libitum and allowed to acclimate for one week before starting experiments.

### 2.2. Preparation of Liposomes

Large unilamellar liposomes composed of egg PC (Nacalai tesque, Kyoto, Japan) and ATTO 740 DOPE (ATTO-TEC GmbH, Siegen, Germany), PE 18:0/22:6 (Avanti Research, Birmingham, AL, USA), PC 18:0/22:6 (Avanti Research, Birmingham, AL, USA), PE P-18:0/22:6 (Avanti Research, Birmingham, AL, USA), or PC P-18:0/22:6; Avanti Research, Birmingham, AL, USA) were prepared via the extrusion method [17]. Briefly, lipids were dissolved in chloroform, and a thin lipid film was obtained by evaporating the lipid–chloroform solution. The film was subsequently hydrated with saline to obtain liposomes (10 mg/mL total phospholipids): egg PC (control), egg PC/PC 18:0/22:6 (4/1: the ratio of the weight of egg PC to that of PC 18:0/22:6 is 4 to 1), egg PC/PC P-18:0/22:6 (4/1), egg PC/PE 18:0/22:6 (4/1), and egg PC/PE P-18:0/22:6 (4/1). After five rounds of freezing and thawing, the lipid suspension was extruded through a polycarbonate filter with 100 nm pore size.

### 2.3. Organ Imaging

Control liposomes or liposomes containing ATTO 740 DOPE (1 mL/kg body weight) were injected into the mouse tail vein as previously described [18,19]. After 3 h, mice were anesthetized by 2% isoflurane inhalation in air and scanned using a Newton 7.0 in vivo imaging system (Vilber-Lourmat, Collégien, France) in order to detect the uptake of fluorescent DOPE in various organs. At 6 h after injection, mice were euthanized, and the brain was harvested for fluorescence detection using the same Newton 7.0 in vivo imaging system [19]. The fluorescent signal intensity of the frontoparietal region in the brain was computed and expressed using arbitrary units (a.u.).

### 2.4. Liposome Injections for Behavioral Tests

Rats were randomly assigned to receive liposomes with egg PC (n = 9) or liposomes with egg PC plus PC 18:0/22:6 (n = 9), PE 18:0/22:6 (n = 9), PC P-18:0/22:6 (n = 9), or PE P-18:0/22:6 (n = 9) through tail vein injection (1 mL/kg body weight) at 9 weeks of age, and examined in the OF, NOR, and EPM tests [18,20]. Subsequently, the marble-burying test was performed in randomly selected rats receiving liposomes with egg PC (n = 6) or liposomes with egg PC plus PC 18:0/22:6 (n = 6), PE 18:0/22:6 (n = 6), PC P-18:0/22:6 (n = 6), or PE P-18:0/22:6 (n = 6).

### 2.5. Behavioral Tests

**Figure 1:**
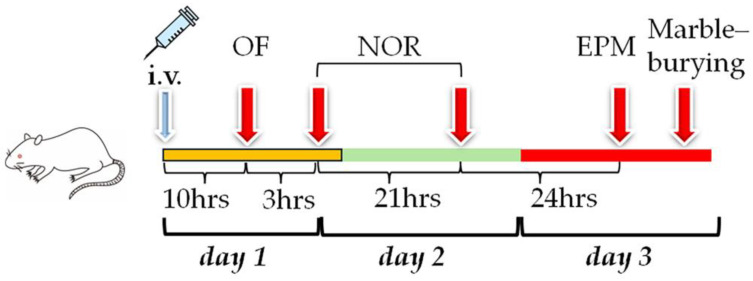
Experimental schedule. Liposomes were injected 10 hours before the open field (OF) test. The OF test was conducted on day 1, followed by the novel object recognition (NOR) test (days 1 and 2), EPM test, and marble-burying test (day 3).

The estrous cycle affects the behavior, as reflected in several behavioral tests. Marble-burying behavior is enhanced at metestrus and decreased at the proestrus [21], while anxious-like behavior in the elevated plus maze is enhanced in the late diestrus phase of the estrous cycle [22]. Thus, male rats were used in this study to explicitly examine the effect of each phospholipid on their behavior. The level of PE P-18:0/22:6 was still elevated in the prefrontal cortex at four days after injection with liposomes incorporating PE P-18:0/22:6 in our previous study [18], and therefore, all the behavioral tests were conducted within three days after liposome injection in this study (Figure 1).

#### 2.5.1. OF Test

Spontaneous locomotor activity was evaluated 10 h after liposome injection (termed Day 1; see Figure 1). Rats were placed individually in a gray chamber measuring 100 cm in diameter and 50 cm in wall height [18]. The OF arena was illuminated from above by 7 lux light. The arena was divided into central and peripheral regions, such that the areas of the central and peripheral regions were the same. The total distance traveled, times spent in the central and peripheral regions, and the frequency of crossings across the borders between the 25 areas (Figure 4F) were quantified automatically over 5 min [19].

#### 2.5.2. NOR Test

The NOR task was completed over two consecutive days in the same arena as used for the OF test, according to a previously described method [23,24] with modifications. (Figure 1). Briefly, rats explored two identical objects (two 500 mL cylindrical water bottles measuring 10 cm in diameter) placed in the arena for 10 min on Day 1, three hours after the acclimation of the rats to the arena during the OF test, and were then returned to their home cages. For the object recognition test on Day 2, one of the two objects was replaced with a new one (a 500 mL square water bottle), and the tested rats were again allowed to explore both objects for 10 min [19]. The times spent exploring the replaced (novel) object and the familiar object were recorded. In this study, time spent with the nose within 7 cm of the object and the head facing toward the object (± 45°) was defined as exploration time [25]. A discrimination ratio (DR) was then calculated to quantify preferential exploration of the novel item compared to the familiar one [26]:

DR = (new object exploration time-old object exploration time)/(total exploration time)

To avoid discrimination of the objects based on odor, both the arena and objects were thoroughly wiped with 10% ethanol before and after each trial.

#### 2.5.3. EPM Test

Anxiety-like behavior was examined in the EPM on Day 3 (Figure 1) [19,20]. The EPM consisted of four arms constructed of gray polyvinyl chloride meeting at a central platform, two closed arms (50 × 10 cm) with 40 cm-high walls, and two open arms (50 × 10 cm) with no side walls. Rats were placed in the central square platform (10 × 10 cm) facing an open arm, and their behavior was recorded for 5 min. The central platform was illuminated at 9 lux, the open arms at 8 lux, and the closed arms at 7 lux. The times spent in the central square platform, open arms, and closed arms were measured.

#### 2.5.4. Marble-Burying Test

To evaluate compulsive-like behaviors, the marble burying test was conducted in standard polycarbonate rat cages (27 cm × 44 cm × 19 cm), according to a previous study [27] with modifications (Figure 1). Fresh, unscented rat bedding material was added to each cage to a depth of 5 cm. Black glass marbles (15 mm diameter, 5.2 g in weight) were gently placed on the surface of the bedding in 5 rows of 4. A rat was placed in the cage containing the marbles and allowed to remain in the cage for 10 min. After returning to the home cage, the number of marbles completely covered by bedding was counted.

### 2.6. Statistics

All the statistical analyses were conducted using JMP Pro 17.0 (SAS Institute Inc., Cary, NC, USA). *p* < 0.05 (corrected for multiple comparisons) was considered statistically significant for all tests. Data are presented as mean ± standard error or deviation, as indicated. Hsu’s MCB test was used to compare all behavioral measures in the OF, NOR, EPM, and marble-burying tests between each group to either the group with the largest or the smallest mean. To examine the behavioral effects conferred by the hydrophilic moiety (choline or ethanolamine) and the chemical bond at *sn*-1 (ester or vinyl–ether), behavioral metrics were compared using two-way ANOVA.

## 3. Results

### 3.1. Incorporation of DOPE into the Mouse Brain

**Figure 2:**
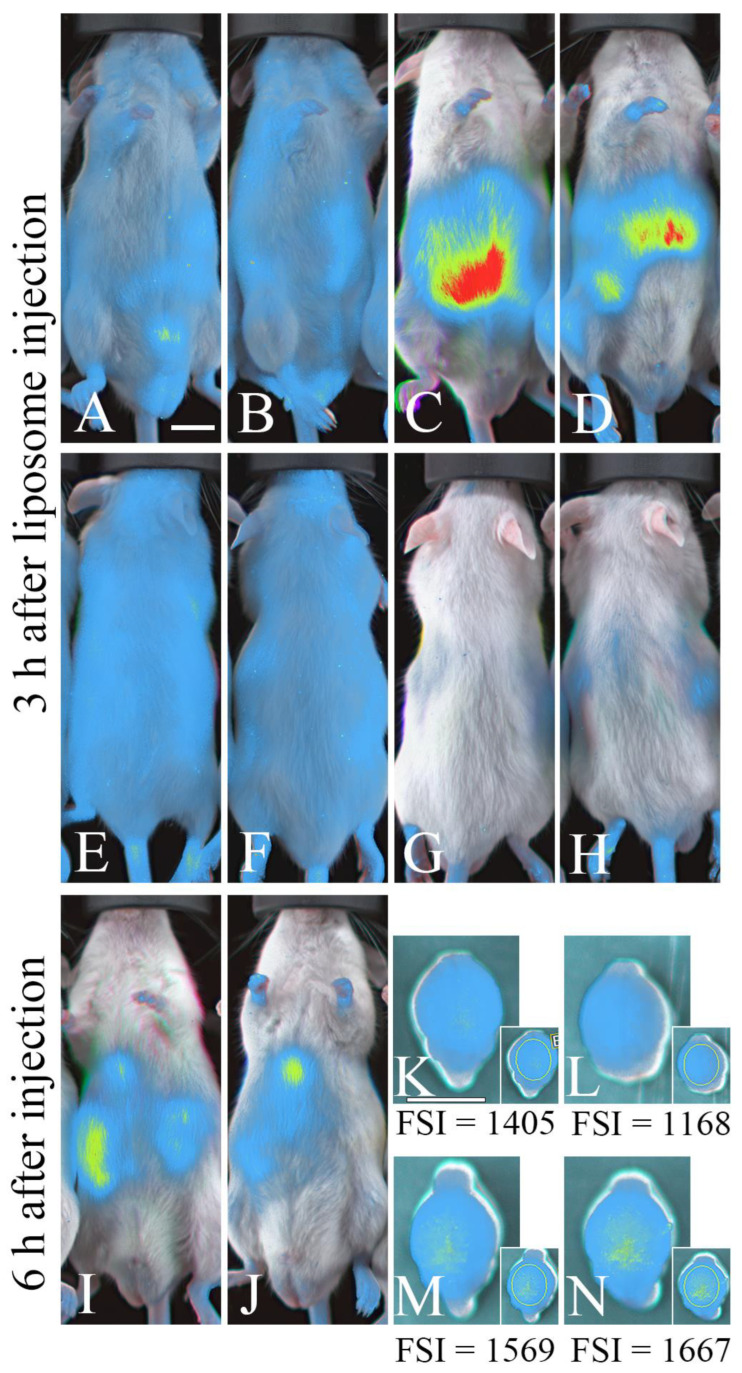
Injected phospholipids accumulate within the brain, as revealed by in vivo fluorescence imaging. (**A**–**H**) Fluorescence images of the ventral (**A**–**D**) and dorsal (**E**–**H**) sides of the body at 3 hours after fluorescent or control liposome injection. (**I**–**N**) Fluorescence images of the ventral side of the body (**I**,**J**) and the dorsal side of the isolated cerebrum (**K**–**N**) 6 hours after liposome injection. (**A**,**B**,**E**,**F**,**K**,**L**) Control mice; (**C**,**D**,**G**,**H**,**I**,**J**,**M**,**N**) liposome-injected mice. Scale bar = 10 mm. FSI: Mean fluorescent signal intensity/pixel (a.u.) in the frontoparietal regions within the yellow ellipse (**K**–**N**).

We used mice to examine whether or not phospholipids can be incorporated into the brain using liposomes, as the body wall is thinner and the brain size is smaller in mice than in rats, and, therefore, small amounts of injected fluorescent phospholipids can be clearly detected. Mouse whole-body and brain scans were conducted using an in vivo imaging system after the intravenous injection of liposomes containing ATTO 740-labeled dioleoylphosphatidylethanolamine (ATTO 740 DOPE, or ATTO 740 PE 18:1/18:1), in order to assess the uptake of liposome contents (DOPE). At 3 h after injection, mice receiving ATTO 740 DOPE but not control mice (receiving liposomes composed of egg PC) exhibited strong fluorescence in the abdominal region, with a region of particularly high intensity appearing to outline the liver (Figure 2A–D). However, little fluorescence was detected in the head (Figure 2E–H). At 6 h post-injection, abdominal fluorescence was weaker (Figure 2I,J), but fluorescence was detected in the frontoparietal region of the cerebrum following removal from the skull (Figure 2K–N), although fluorescence in the brain was not detected through the skull (presumably due to the light scattering by the skull) [28]. Our previous study showed that fluorescence was detected in the brain at 3 h after injection of the liposomes, including ATTO 740 DOPE [19]; thus, DOPE can be incorporated into the brain within 3 h of injection, and some of the DOPE still remained at 6 h after injection.

### 3.2. Behavioral Changes Induced by the Injection of Phospholipid Liposomes

#### 3.2.1. Locomotor Activities

**Figure 3:**
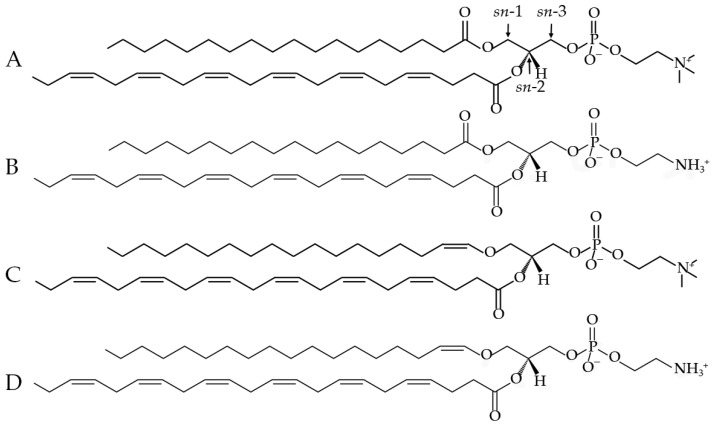
Chemical structures of the phospholipids injected into the rats. (**A**). PC 18:0/22:6; (**B**). PE 18:0/22:6; (**C**). PC P-18:0/22:6; (**D**). PE P-18:0/22:6. Plasmalogens (**C**,**D**) have a vinyl–ether bond instead of an ester bond at the *sn*-1 position.

**Figure 4:**
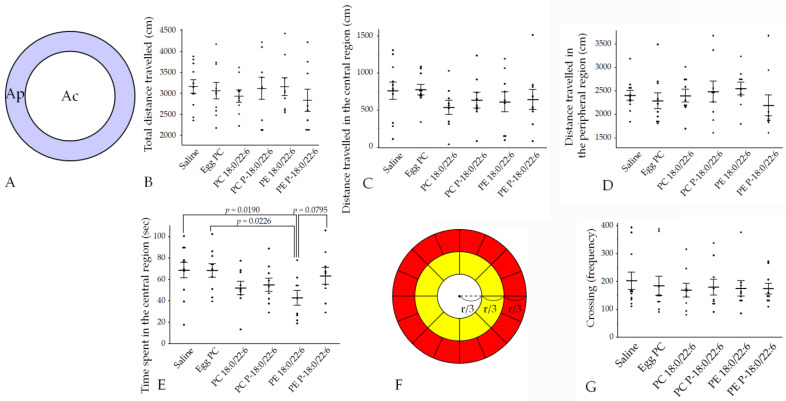
Injection of liposomes containing PE 18:0/22:6 reduced exploration in the open field. (**A**) The central and peripheral regions. The area of the central region (Ac, white area) is the same as that of the peripheral region (Ap, purple area). (**B**) Total distance traveled. (**C**) Distance traveled in the central region. (**D**) Distance traveled in the peripheral region. (**E**) Time spent in the central region. (**F**) Twenty-five equal areas for counting crossings. The radius of the smallest circle is 1/3 the radius of the largest circle (r), and the radius of the medium-sized circle is 2/3 the radius of the largest circle. The yellow region is divided into 8 equal areas, and the red region is divided into 16 equal areas. (**G**) Number of area crossings. Bars presented as mean ± SE. The rat numbers in each group were as follows: saline (n = 11), egg PC (n = 10), PC 18:0/22:6 (n = 9), PE 18:0/22:6 (n = 9), PC P-18:0/22:6 (n = 9), and PE P-18:0/22:6 (n = 9). Differences were evaluated using Hsu’s MCB test.

The total distance traveled, the distance traveled in the central and peripheral regions, and the frequency of the crossings in the open field (OF) tests did not differ among the saline, egg PC, PC 18:0/22:6, PE 18:0/22:6, PE P-18:0/22:6, and PC P-18:0/22:6 groups (Figure 3 and Figure 4, Table S1).

However, PE 18:0/22:6 group mice spent significantly less time in the central region compared to the saline and egg PC groups (*p* = 0.0182 and *p* = 0.0226), according to Hsu’s multiple comparisons with the best (MCB) test (Figure 4, Table S1). Additionally, time spent in the central region tended to be longer in PE P-18:0/22:6 groups compared to PE 18:0/22:6 (*p* = 0.0795) (Figure 4, Table S1).

There was no interaction effect between the specific hydrophilic head group (choline or ethanolamine) and the chemical bond at the *sn*-1 position (ester or a vinyl–ether) on OF behaviors (all *p* > 0.05 by two-factor ANOVA); however, substitution of an ester bond with a vinyl–ether bond tended to increase the time spent in the central region (*p* = 0.094 for the main effect of a bond at *sn*-1; Table 1) in line with the results of Hsu’s MCB test.

#### 3.2.2. Novel Object Recognition Memory

**Figure 5:**
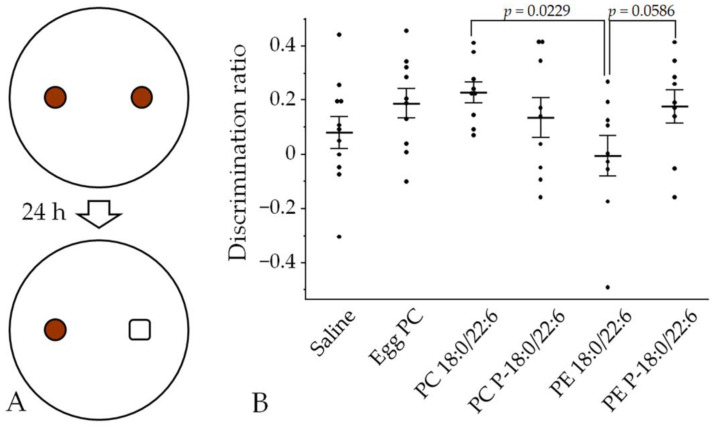
Effects of liposome injection on object recognition memory, as expressed by the discrimination ratio (DR). (**A**) Rats explored two cylindrical bottles placed in the arena for 10 min and, 24 h later, one of the two objects was replaced with a new (square) bottle. Then, the tested rats were again allowed to explore both objects for 10 min. (**B**) Bars presented as mean ± SE. The rat numbers in each group were as follows: saline (n = 11), egg PC (n = 10), PC 18:0/22:6 (n = 9), PE 18:0/22:6 (n = 9), PC P-18:0/22:6 (n = 9), and PE P-18:0/22:6 (n = 9). Differences were evaluated using Hsu’s MCB test.

A NOR test was performed to examine the effects of phospholipid injections on associative memory. The discrimination ratio (DR) increased significantly in the PC 18:0/22:6 group compared with the PE 18:0/22:6 group, while DR tended to increase in the PE P-18:0/22:6 group compared with the PE 18:0/22:6 group (Figure 5, Table S2). Additionally, two-factor ANOVA revealed a significant interaction effect between the hydrophilic head group (choline or ethanolamine) and chemical bonds at the *sn*-1 position (ester or vinyl–ether) on DR (*p* = 0.039; Table 1). There was a significant simple main effect between PC and PE with the ester bond in DR (*p* = 0.014; Table 2). Similarly, a trend of the difference in DR between an ester and a vinyl–ether bond in PE was observed (*p* = 0.051; Table 2). These results suggest that choline at *sn*-3 in the glycerophospholipids and a vinyl–ether bond at *sn*-1 in PE are involved in the enhancement of recognition memory with respect to the original object configuration.

#### 3.2.3. Anxiety-like and Compulsive-like Behaviors

**Figure 6:**
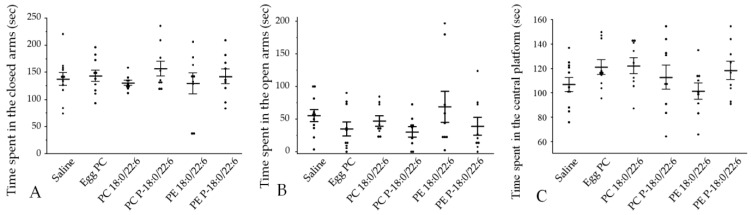
Effects of liposome injection on anxiety-like behavior in the elevated plus maze. (**A**) Time spent in the closed arms. (**B**) Time spent in the open arms. (**C**) Time spent in the central platform. Bars presented as mean ± SE. The rat numbers in each group were as follows: saline (n = 11), egg PC (n = 10), PC 18:0/22:6 (n = 9), PE 18:0/22:6 (n = 9), PC P-18:0/22:6 (n = 9), and PE P-18:0/22:6 (n = 9). Differences were evaluated using Hsu’s MCB test.

Elevated plus maze and marble burying tests were performed to examine anxiety-like and compulsive-like behaviors. There were no significant differences in time spent in the open arms, closed arms, and central platform of the EPM among the saline, egg PC, PC 18:0/22:6, PE 18:0/22:6, PC P-18:0/22:6, and PE P-18:0/22:6 groups (*p* > 0.05 by Hsu’s MCB test; Figure 6, Table S3).

**Figure 7:**
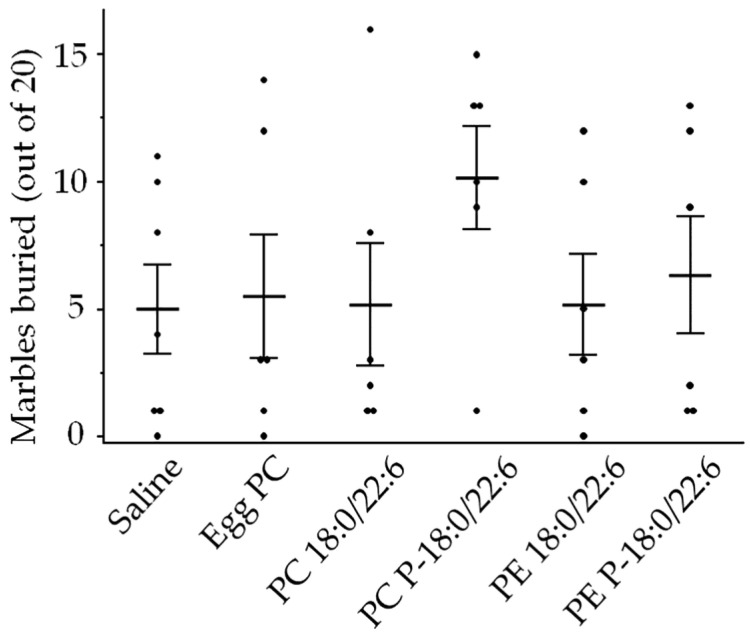
Effects of liposome injection on compulsion-like behavior, assessed according to the number of marbles buried. Bars presented as mean ± SE. The rat numbers in each group were as follows: saline (n = 11), egg PC (n = 6), PC 18:0/22:6 (n = 6), PE 18:0/22:6 (n = 6), PC P-18:0/22:6 (n = 6), and PE P-18:0/22:6 (n = 6). Differences were evaluated using Hsu’s MCB test.

There were also no significant differences in the number of marbles buried among the groups (Figure 7, Table S4). Furthermore, the two-factor ANOVA revealed no significant interaction effect between the hydrophilic head group and chemical bonds at the *sn*-1 position on either EPM or marble-burying behavior (Table 1).

## 4. Discussion

### 4.1. Effects of Phospholipid Moieties on Novel Object Recognition Memory

Previous studies have suggested that PlsEtn deficiency and exogenous administration can modulate brain functions and behavior in rodents and humans. Han and coworkers reported that PlsEtn was markedly reduced in cerebral and cerebellar white matter and that a PlsEtn deficiency in gray matter was correlated with the severity of clinical dementia [6]. In Parkinson’s disease and dementia with Lewy bodies patients, plasmalogen concentrations were reduced in the lipid rafts from the frontal cortex [29]. Conversely, a plasmalogen-enriched diet containing PlsEtn, PlsCho, sphingomyelin, and other phospholipids was found to increase Pl content in the hippocampus and to accelerate brain-derived neurotrophic factor (BDNF) signaling in mice by promoting tyrosine kinase receptor B (TrkB) expression in the lipid rafts of the hippocampus, and concomitantly to enhance learning and memory in the NOR test [8]. Eicosapentaenoic acid (EPA)-enriched PE and the combination of Chlorella and ascidian PlsEtn activated BDNF/TrkB/cAMP response element-binding protein (CREB) signaling, which has an important role in neurogenesis, synaptic plasticity, and cognition, in the hippocampus [30,31]. Additionally, a mixture of three ethanol amine plasmalogens, one choline plasmalogen, and ceramide alpha-hydroxy fatty-acid-sphingosine improved memory function by regulating hippocampal neurogenesis by activating the Wnt/β-catenin pathway in a mouse model of Alzheimer’s disease [32]. Collectively, these studies suggest that the enrichment of plasmalogens in the hippocampus can improve learning and memory through BDNF/TrkB/CREB signaling and/or the Wnt/β-catenin pathway. However, the question of which intramolecular structures in the phospholipids are able to improved memory has not yet been definitively answered.

In our study, we found that the substitution of ethanolamine with choline restored the recognition memory. Additionally, the substitution of an ester bond with a vinyl–ether bond at *sn*-1 in PE tended to enhance recall in the NOR. The intramolecular hydrogen bonds (amino-phosphate hydrogen bonds) of the hydrophilic moiety in the PE [33] and the vinyl–ether bonds being less capable of forming hydrogen bonds with water molecules than the ester bond [12,34], cooperatively increasing the packing at the glycerol backbone region in the PlsEtn due to the hydrophobic effect and forms a hexagonal II phase. The increased packing in PlsEtn leads to the formation of a more ordered acyl chain in the phospholipids and results in a more rigid bilayer [12]. In contrast, the surface of a PC bilayer is hydrophilic compared to the surface of a PE bilayer [33]; therefore, PC forms a less rigid lamellar bilayer structure compared to PE. The shift in the bilayer rigidity may play a role in ameliorating recognition memory in PC and PlsEtn injections through modulation of the intracellular signaling and neurotransmission. This hypothesis may partially explain the finding that PC with a vinyl–ether bond did not restore recall compared to PE as follows: phosphocholine may counteract the effect of a vinyl–ether bond on membrane rigidity, therefore, the rigidity of a PC bilayer containing a vinyl–ether bond may be close to that of a PE bilayer. Thus, the hydrophilic moiety and a vinyl–ether bond may be involved in recall through alterations in the membrane rigidity due to the hydrophobic effect. On the other hand, previous studies have demonstrated that omega-3 fatty acids such as DHA and EPA can improve learning and memory in non-cognitively impaired participants and patients with mild cognitive impairment or dementia [35]. In rats, blood lysophosphatidylcholine-DHA (LPC-DHA), which is actively transported into the endothelium of the BBB through the symport major facilitator superfamily domain-containing protein 2A (Mfsd2a), passes through the BBB more effectively than PC-DHA or DHA alone. In wild-type rats, dietary DHA from LPC-DHA enriched brain DHA more effectively than dietary PC-DHA; as a result, only dietary LPC-DHA improved spatial learning in the Morris water maze test [36,37]. These studies suggest that the incorporation of DHA into phospholipids can improve memory deficits when in the appropriate structural configuration; however, DHA at the *sn*-2 position in PC and PE examined in our study, at least, had no effect on memory.

### 4.2. Effects of Brain Phospholipid Moieties on Locomotor Activity, Anxiety, and Compulsive-like Behavior

Rats injected with liposomes containing PE 18:0/22:6 also exhibited reduced time spent in the central region of the OF, but these liposomes did not influence anxiety-like behavior in the elevated plus maze or compulsive-like behavior in the marble burying test. Therefore, the decreased time spent in the central region of the OF instead suggests reduced exploratory drive [20,38]. Furthermore, this reduced exploration may be offset by the substitution of the ester bond with a vinyl–ether bond at *sn*-1, according to two-way ANOVA. In mice with traumatic right parietal cortex injury, lower PE, higher triacylglycerol, lower hexosylceramide, and lower PE with ether-linkage in the perilesional and subregional areas of ipsilateral cortex were correlated with non-goal-directed nighttime hyperactivity (nighttime “corner visits with nose pokes but without licks”) as measured using the IntelliCage system [39]. Results of our fluorescence tracer study suggested that PE was incorporated into the mouse frontoparietal region and that PE 18:0/22:6 reduced exploration in rats. Thus, elevated PE in the frontoparietal region may reduce exploratory behavior. In accordance with this finding, a valproic acid-induced autistic mouse model with elevated PE in the cortex, in addition to the hippocampus, thalamus, hypothalamus, cerebellum, and brain stem [40] also spent less time in the central region of the OF [41], as well as displaying reduced exploratory activity [42] compared to control mice.

Furthermore, the substitution of an ester bond with a vinyl–ether bond tended to increase the time spent in the central region. Our previous study revealed that PE P-18:0/22:6, which has a vinyl–ether bond at *sn*-1, reversed the decline in central region time with age [18]. In healthy humans, a gradual linear decline in brain ethanolamine plasmalogen concentration starts at 30 years old [43]. Similarly, age-dependent reductions in ethanolamine plasmalogen, including PE 18:0/22:6, have been observed in the frontal cortex of mice, while PE (with an ester bond at *sn*-1) did not decrease with age [44]. Therefore, the balance between ester-linked PE and vinyl–ether-linked PE in the cortex may have a critical role in modulating exploratory behavior. In contrast, DHA at *sn*-2 had no impact on anxiety-like behavior or compulsive-like behavior regardless of the hydrophilic moiety and the bond at *sn*-1. The specific hydrophilic moiety as the head group and the chemical bond at *sn*-1 may be critical to the PE-dependent modulation of exploratory behavior.

### 4.3. Limitations and Future Prospects

There are some limitations to our study. Our present study lacks data involving the intake and distribution process of injected phospholipids and the activating or inhibiting pathway that led to the recognition of memory and exploratory behaviors. Furthermore, the physicochemical interactions between phospholipid moieties and biomolecules remain relatively unknown. However, we provided a clue to design effective phospholipids for memory improvement and controlling exploratory behaviors. Further studies elucidating the associations between the phospholipid function and the chemical structures at *sn*-1, *sn*-2, and *sn*-3 positions are warranted.

## 5. Conclusions

An ester bond at *sn*-1 of PE 18:0/22:6 may inhibit exploratory behavior in male rats, whereas substitution of this ester bond with a vinyl–ether bond may restore exploratory behavior. In addition to this substitution, the substitution of ethanolamine with choline in phospholipids integrating DHA may also improve recognition memory. The form of the *sn*-1 bond connecting the glycerol backbone to the FA and the hydrophilic moiety at *sn*-3 may have a critical role in modulating behavior and cognition.

## Figures and Tables

**Table 1:** Effects of brain phospholipid moieties on rat behaviors.

Behavioral Tests		*p*-Value		
		Two-Way ANOVA		
		*sn*-1	Hydrophilic Moiety (HM)	*sn*-1 × HM
OFT	Total distance traveled	0.756	0.900	0.268
	Distance traveled in the central region	0.589	0.733	0.789
	Distance traveled inthe peripheral region	0.469	0.709	0.236
	Crossings across the border between areas	0.845	0.986	0.812
	Time spent in the central region	0.094	0.952	0.205
NOR	Discrimination ratio	0.484	0.141	0.039 *
EPM	Time spent in the open arms	0.125	0.310	0.666
	Time spent in the closed arms	0.170	0.585	0.626
	Time spent in the central platform	0.622	0.336	0.098
MB	Number of marbles buried	0.175	0.393	0.393

OF: open field test, NOR: novel object recognition test, EPM: elevated plus maze test, MB: marble-burying test. * *p* < 0.05.

**Table 2:** The simple main effects test according to the discrimination ratio.

Simple Main Effects of *sn*-1 Bonds Within Phospholipid Species			Simple Main Effects of Phospholipid Species Within *sn*-1 Bonds		
Phospholipid Species	*sn*-1 Bonds	*p* Value	*sn*-1 Bonds	Phospholipid Species	*p* Value
PC	ester vs. vinyl–ether	0.313	ester	PC vs. PE	0.014
PE	ester vs. vinyl–ether	0.051	vinyl–ether	PC vs. PE	0.649

## Data Availability

Data will be available upon request.

## Supplementary Materials

The following supporting information can be downloaded at https://www.mdpi.com/article/10.3390/neurosci5040037/s1, Table S1: Open field test; Table S2: Novel object recognition test; Table S3: Elevated plus maze test; Table S4: Marble burying test.
