# Supplementary material for: Ethanolamine and Vinyl–Ether Moieties in Brain Phospholipids Modulate Behavior in Rats
Source: NeuroSci. 2024 Nov 4;5(4):509–22. doi: 10.3390/neurosci5040037 (PMC11587438; doi:10.3390/neurosci5040037)
Supplement: Supplementary file 1 [file neurosci-05-00037-s001.zip › TableS1.pdf]

Table S1 Open field test

		N	Mean	SD	*p*-value (Hsu's MCB)
Total distance travelled	Saline	11	3167.1	529.0	0.366
	Egg PC	10	3065.4	631.7	0.526
	PC 18:0/22:6	9	2935.6	440.9	0.712
	PE 18:0/22:6	9	3162.2	643.9	0.441
	PC P-18:0/22:6	9	3119.9	784.6	0.775
	PE P-18:0/22:6	9	2835.9	785.9	0.909
Crossing	Saline	11	202.8	102.6	0.501
	Egg PC	10	185.0	108.6	0.699
	PC 18:0/22:6	9	169.1	73.7	0.865
	PE 18:0/22:6	9	175.3	82.3	0.779
	PC P-18:0/22:6	9	179.9	85.8	0.739
	PE P-18:0/22:6	9	174.6	58.7	0.786
Distance travelled in the central region	Saline	11	760.6	387.2	0.245
	Egg PC	10	775.6	220.2	0.218
	PC 18:0/22:6	9	538.1	283.0	0.931
	PE 18:0/22:6	9	611.0	403.9	0.661
	PC P-18:0/22:6	9	634.9	316.7	0.595
	PE P-18:0/22:6	9	643.7	403.1	0.571
Distance travelled in the peripheral region	Saline	11	2406.5	346.2	0.468
	Egg PC	10	2289.8	536.1	0.691
	PC 18:0/22:6	9	2397.6	405.6	0.484
	PE 18:0/22:6	9	2551.2	413.3	0.224
	PC P-18:0/22:6	9	2484.9	668.4	0.325
	PE P-18:0/22:6	9	2192.2	670.5	0.924
Time spent in the central region	Saline	11	68.6	24.2	0.0190
	Egg PC	10	68.4	19.8	0.0226
	PC 18:0/22:6	9	52.0	18.6	0.445
	PE 18:0/22:6	9	42.8	20.6	0.979
	PC P-18:0/22:6	9	55.0	18.6	0.317
	PE P-18:0/22:6	9	63.4	23.5	0.0795

p-value: vs group with the smallest mean
