# Supplementary material for: Ethanolamine and Vinyl–Ether Moieties in Brain Phospholipids Modulate Behavior in Rats
Source: NeuroSci. 2024 Nov 4;5(4):509–22. doi: 10.3390/neurosci5040037 (PMC11587438; doi:10.3390/neurosci5040037)
Supplement: Supplementary file 1 [file neurosci-05-00037-s001.zip › TableS2.pdf]

Table S2 Novel object recognition test

	Phospholipids	N	Mean	SD	*p*-value (Hsu's MCB)
Discrimination ratio	Saline	11	0.0819	0.196	0.421
	Egg PC	10	0.188	0.172	0.0586
	PC 18:0/22:6	9	0.229	0.117	0.0229
	PE 18:0/22:6	9	-0.005	0.227	0.984
	PC P-18:0/22:6	9	0.137	0.219	0.187
	PE P-18:0/22:6	9	0.178	0.184	0.0811

p-value: vs group with the smallest mean
