# Supplementary material for: Ethanolamine and Vinyl–Ether Moieties in Brain Phospholipids Modulate Behavior in Rats
Source: NeuroSci. 2024 Nov 4;5(4):509–22. doi: 10.3390/neurosci5040037 (PMC11587438; doi:10.3390/neurosci5040037)
Supplement: Supplementary file 1 [file neurosci-05-00037-s001.zip › TableS3.pdf]

Table S3 Elevated plus maze test

	Phospholipids	N	Mean	SD	*p*-value (Hsu's MCB)
Time spent in the closed arms	Saline	11	137.7	39.5	0.691
	Egg PC	10	143.7	33.1	0.534
	PC 18:0/22:6	9	130.7	13.8	0.815
	PE 18:0/22:6	9	129.8	57.8	0.841
	PC P-18:0/22:6	9	157.0	41.7	0.233
	PE P-18:0/22:6	9	142.5	40.6	0.562
Time spent in the open arms	Saline	11	55.4	30.2	0.264
	Egg PC	10	35.1	33.1	0.752
	PC 18:0/22:6	9	47.2	23.8	0.461
	PE 18:0/22:6	9	68.9	70.9	0.0832
	PC P-18:0/22:6	9	30.3	24.0	0.895
	PE P-18:0/22:6	9	39.1	41.5	0.654
Time spent in the central platform	Saline	11	106.9	19.1	0.631
	Egg PC	10	121.2	19.2	0.0956
	PC 18:0/22:6	9	122.1	19.7	0.0860
	PE 18:0/22:6	9	101.3	20.1	0.948
	PC P-18:0/22:6	9	112.8	30.0	0.362
	PE P-18:0/22:6	9	118.4	22.3	0.166

p-value: vs group with the smallest mean
