# Supplementary material for: Ethanolamine and Vinyl–Ether Moieties in Brain Phospholipids Modulate Behavior in Rats
Source: NeuroSci. 2024 Nov 4;5(4):509–22. doi: 10.3390/neurosci5040037 (PMC11587438; doi:10.3390/neurosci5040037)
Supplement: Supplementary file 1 [file neurosci-05-00037-s001.zip › TableS4.pdf]

Table S4 Marble burying test

	Phospholipids	N	Mean	SD	*p*-value (Hsu's MCB)
Number of marbles buried	Saline	7	5.00	4.619	0.862
	Egg PC	6	5.50	5.958	0.776
	PC 18:0/22:6	6	5.17	5.913	0.814
	PE 18:0/22:6	6	5.17	4.875	0.814
	PC P-18:0/22:6	6	10.17	4.997	0.151
	PE P-18:0/22:6	6	6.33	5.645	0.666

p-value: vs group with the smallest mean
